# Supplementary material for: TCR2HLA: Calibrated inference of HLA genotypes from TCR repertoires enables identification of immunologically relevant metaclonotypes
Source: PLoS Comput Biol. 2026 Jan 16;22(1):e1013767. doi: 10.1371/journal.pcbi.1013767 (PMC12810895; doi:10.1371/journal.pcbi.1013767)
Supplement: S1 Fig — (PDF) [file pcbi.1013767.s008.pdf]

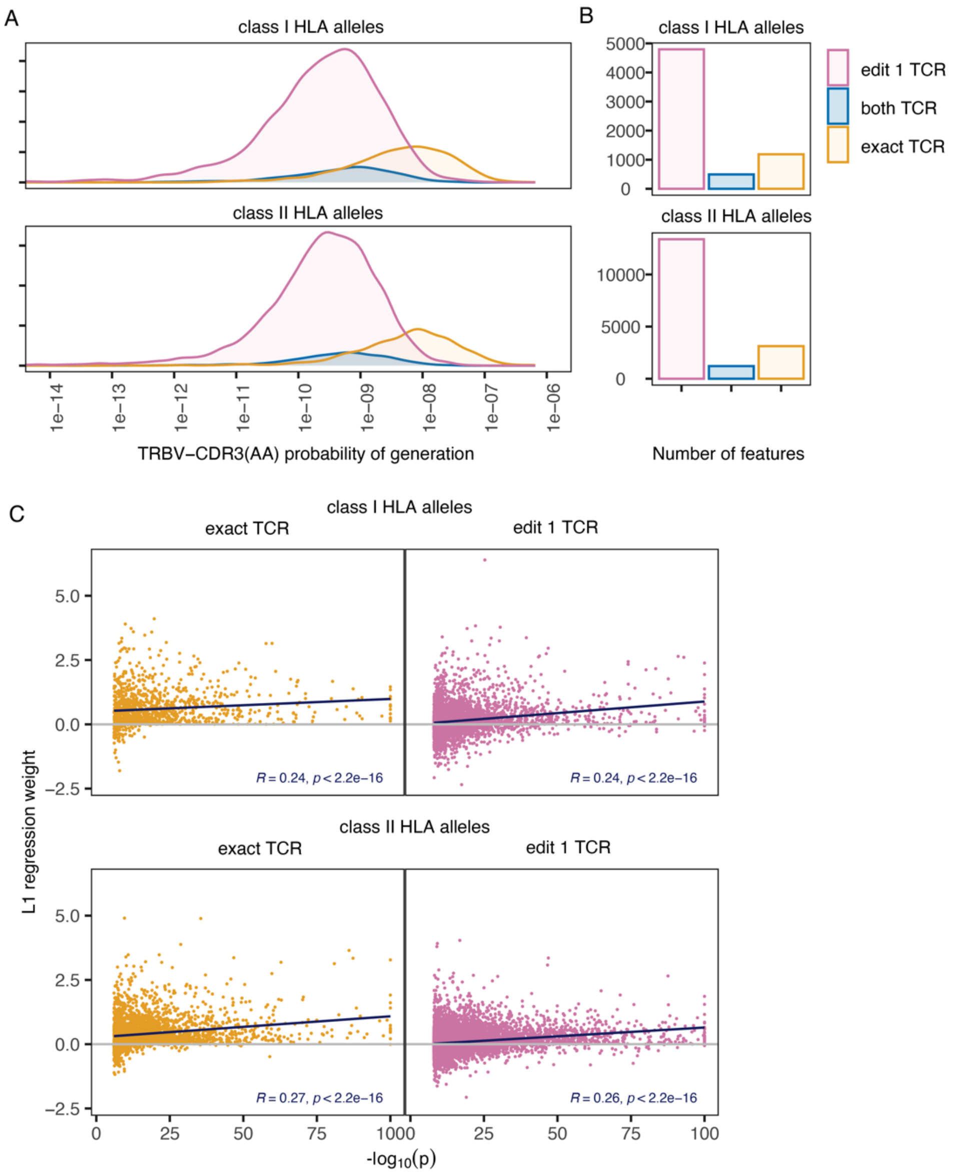

Figure S1. Probability of Generation estimates and L1-regularized feature weights by TCR feature type.

(**A**) Estimated probability of generation (Pgen) of TCRs (TRBV-CDR3AA) associated with common class I or class II HLA alleles.

(**B**) Number of exact or near-exact (edit distance = 1) HLA-associated TCR with non-zero L1-regularized regression weights. TCR features strongly associated with a given HLA allele by exact matching (orange), near-exact matching including single mutational variants (pink), or both (blue) are shown.

(**C**) Correlation between the $-\log_{10}$ p-values for HLA-specific enrichment (Fisher's exact test) of individual TCRs or TCR neighborhoods in the training data and the corresponding regression coefficients estimated from combined features in L1-penalized regressions.
