# Supplementary material for: TCR2HLA: Calibrated inference of HLA genotypes from TCR repertoires enables identification of immunologically relevant metaclonotypes
Source: PLoS Comput Biol. 2026 Jan 16;22(1):e1013767. doi: 10.1371/journal.pcbi.1013767 (PMC12810895; doi:10.1371/journal.pcbi.1013767)
Supplement: S2 Fig — (PDF) [file pcbi.1013767.s009.pdf]

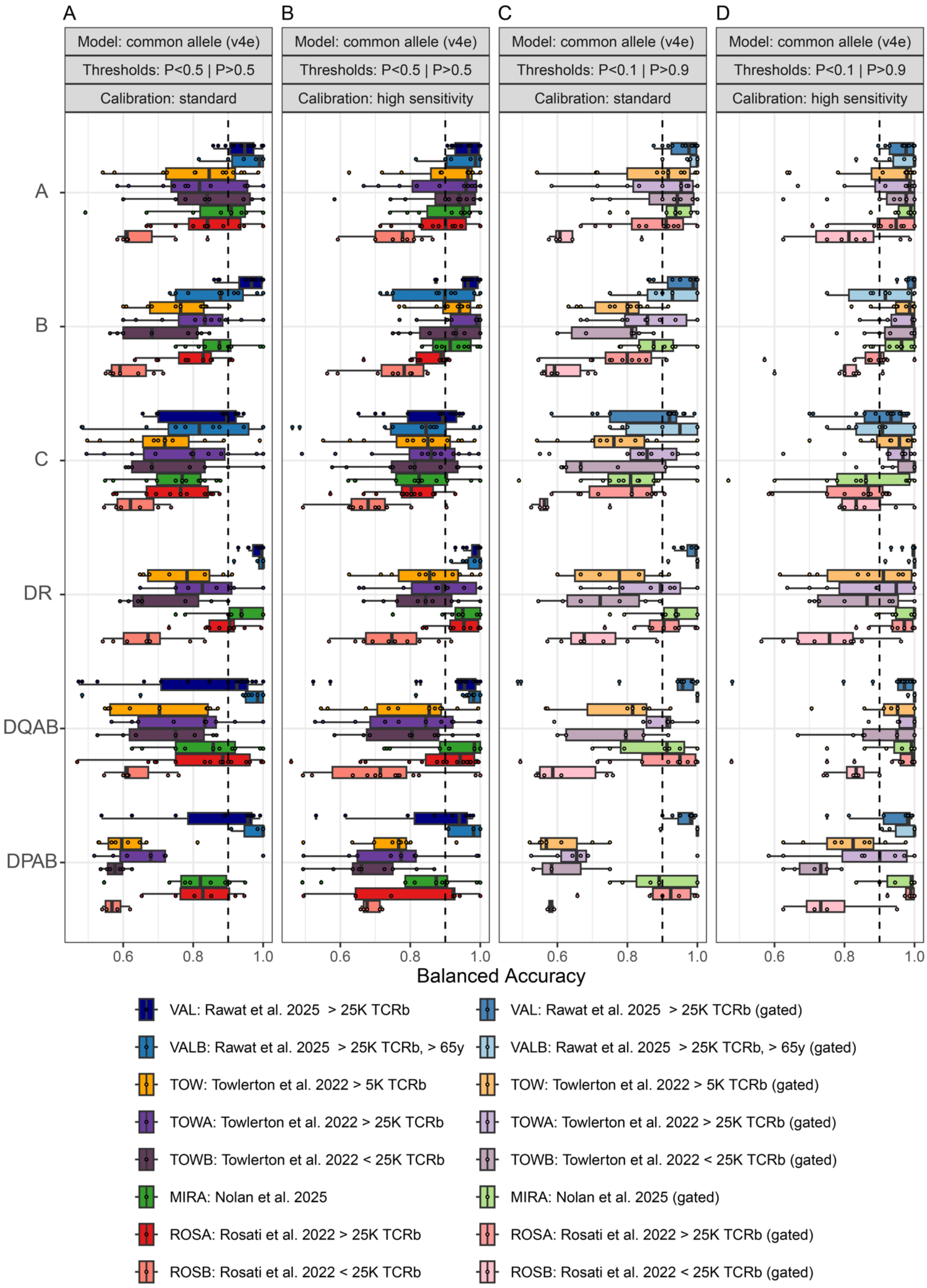

A
B
C
D
Model: common allele (v4e)
Thresholds: P<0.5 | P>0.5
Thresholds: P<0.1 | P>0.9
Calibration: standard
Calibration: high sensitivity
A
B
C
DR
DQAB
DPAB
0.6
0.8
1.0
Balanced Accuracy
VAL: Rawat et al. 2025 > 25K TCRb
VALB: Rawat et al. 2025 > 25K TCRb, > 65y
TOW: Towlerton et al. 2022 > 5K TCRb
TOWA: Towlerton et al. 2022 > 25K TCRb
TOWB: Towlerton et al. 2022 < 25K TCRb
MIRA: Nolan et al. 2025
ROSA: Rosati et al. 2022 > 25K TCRb
ROSB: Rosati et al. 2022 < 25K TCRb
VAL: Rawat et al. 2025 > 25K TCRb (gated)
VALB: Rawat et al. 2025 > 25K TCRb, > 65y (gated)
TOW: Towlerton et al. 2022 > 5K TCRb (gated)
TOWA: Towlerton et al. 2022 > 25K TCRb (gated)
TOWB: Towlerton et al. 2022 < 25K TCRb (gated)
MIRA: Nolan et al. 2025 (gated)
ROSA: Rosati et al. 2022 > 25K TCRb (gated)
ROSB: Rosati et al. 2022 < 25K TCRb (gated)

Figure S2. Balanced accuracy on different validation datasets.

Balanced accuracy was estimated by predicting common HLA alleles across DNA-based (Rawat et al. 2024, Towlerton et al. 2022, Nolan et al. 2025 (MIRA) or RNA-based (Rosati 2022) TCRseq external validation data sets. The standard calibration was based on the full training data. The high sensitivity calibration was based on repeated down sampling of the training data to incorporate an adjustment based on the number of unique clonotypes ranging from 5,000 to 500,000 (see Methods).

(**A**) Standard calibration using a P > 0.5 decision threshold.

(**B**) High sensitivity calibration using P > 0.5 decision threshold.

(**C**) Standard calibration with intermediate confidence predictions (0.1 > P(HLA) < .9) removed.

(**D**) High sensitivity calibration with intermediate confidence predictions (0.1 > P(HLA) < .9) removed.
