# Supplementary material for: TCR2HLA: Calibrated inference of HLA genotypes from TCR repertoires enables identification of immunologically relevant metaclonotypes
Source: PLoS Comput Biol. 2026 Jan 16;22(1):e1013767. doi: 10.1371/journal.pcbi.1013767 (PMC12810895; doi:10.1371/journal.pcbi.1013767)
Supplement: S3 Fig — (PDF) [file pcbi.1013767.s010.pdf]

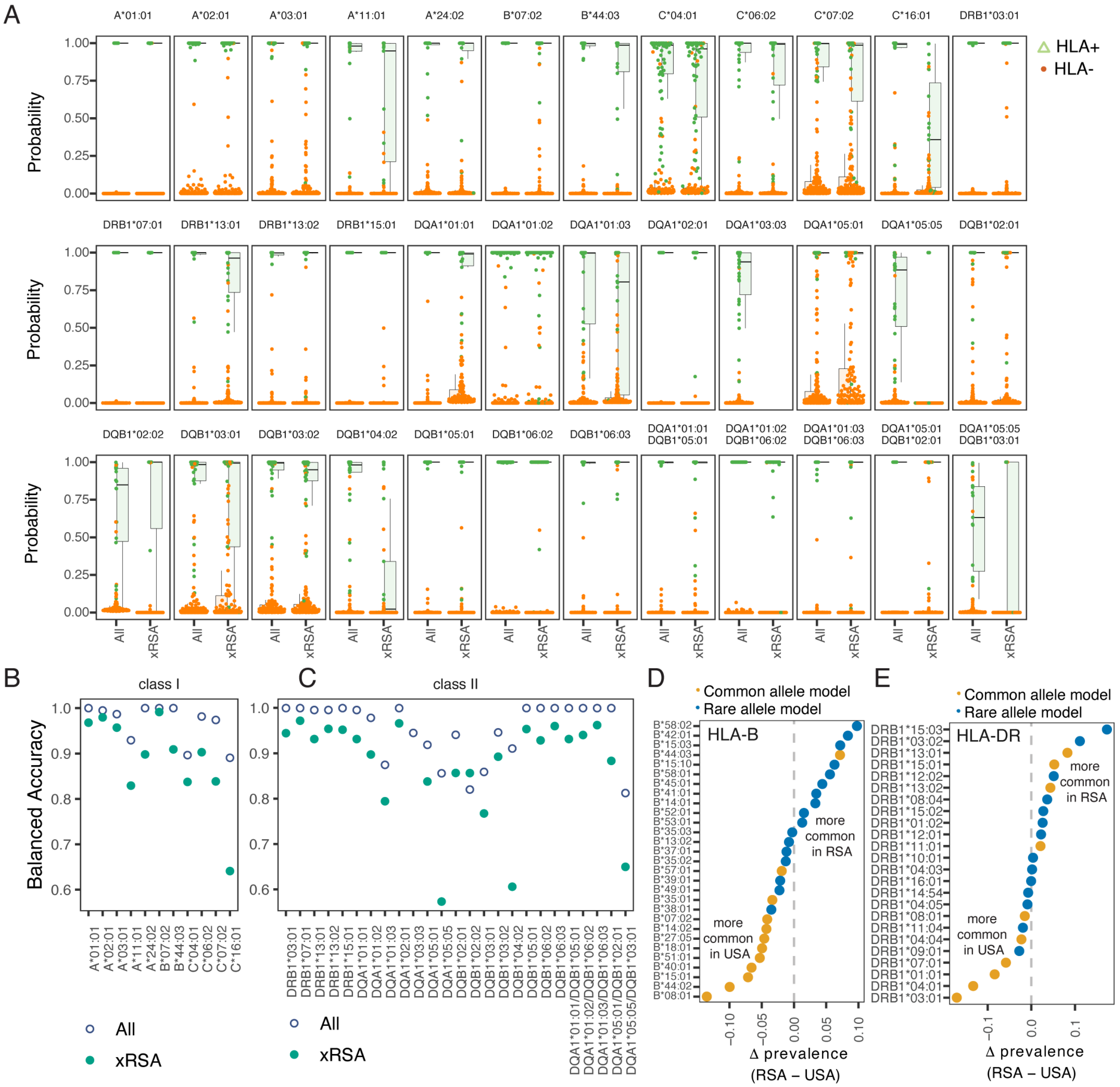

Figure S3. HLA-inference to a geographically distinct population.

(**A**) For repertoires from the Republic of South Africa (RSA) from Musvosvi et al. 2023, calibrated HLA-allele probabilities were generated by models using all training data (All) or after removing RSA samples (xRSA). Results shown for alleles with at least 15 positive cases.

(**B**) Balanced accuracy of predictions of full vs. ablated models for common class I or class II HLA alleles. Results shown for alleles with at least 15 or more positive cases.

(**D**) Difference in allelic prevalence between the RSA and US training data for representative HLA-B.

(**E**) Difference in allelic prevalence between the RSA and US training data for representative HLA-DR.
